# Combination therapy with Olaratumab/doxorubicin in advanced or metastatic soft tissue sarcoma -a single-Centre experience

**DOI:** 10.1186/s12885-020-6551-y

**Published:** 2020-01-29

**Authors:** Jana Käthe Striefler, Franziska Brandes, Alexander Baur, Berit Maria Pfitzner, David Kaul, Daniel Rau, Anne Dörr, Maren Schmiester, Georgios Koulaxouzidis, Lars Bullinger, Sven Märdian, Anne Flörcken

**Affiliations:** 1 Department of Hematology, Oncology, and Tumor Immunology, Charité–Universitätsmedizin Berlin, corporate member of Freie Universität Berlin, Humboldt-Universität zu Berlin, and Berlin Institute of Health, Campus Virchow-Klinikum, Augustenburger Platz 1, 13353 Berlin, Germany; 2 Department of Radiology, Charité–Universitätsmedizin Berlin, corporate member of Freie Universität Berlin, Humboldt-Universität zu Berlin, and Berlin Institute of Health, Campus Virchow-Klinikum, Berlin, Germany; 3 DRK Kliniken Berlin Köpenick, Institut für Pathologie, Berlin, Germany; 4 Department of Radiation Oncology, Charité–Universitätsmedizin Berlin, corporate member of Freie Universität Berlin, Humboldt-Universität zu Berlin, and Berlin Institute of Health, Campus Virchow-Klinikum, Berlin, Germany; 5 Charité–Universitätsmedizin Berlin, corporate member of Freie Universität Berlin, Humboldt-Universität zu Berlin, and Berlin Institute of Health, Centre for Musculoskeletal Surgery, Campus Virchow-Klinikum, Berlin, Germany; 6 Department of Surgery, Charité–Universitätsmedizin Berlin, corporate member of Freie Universität Berlin, Humboldt-Universität zu Berlin, and Berlin Institute of Health, Plastic and Reconstructive Surgery, Campus Virchow-Klinikum, Berlin, Germany; 7 German Cancer Consortium (DKTK), partner site Berlin, and German Cancer Research Center (DKFZ), Heidelberg, Germany

**Keywords:** Soft tissue sarcoma, Doxorubicin, Olaratumab, Platelet-derived growth factor receptor alpha (PDGFRA), Hyperthermia

## Abstract

**Background:**

The antibody targeting platelet-derived growth factor receptor alpha (PDGFRA), olaratumab, was approved in 2016 for metastatic soft tissue sarcoma (STS) in combination with doxorubicin based on promising results of a phase Ib/II trial by the Food and Drug Administration (FDA). However, recently the phase III ANNOUNCE trial could not confirm the additional value of olaratumab in this context.

**Methods:**

Here, in a retrospective analysis we share our single-centre experience with olaratumab/doxorubicin in STS by including *n* = 32 patients treated with olaratumab/doxorubicin between 2016 and 2019.

**Results:**

Median progression-free survival (PFS) in the overall cohort was 3.1 months (range 0.6–16.2). A response [complete remission (CR), partial remission (PR) or stable disease (SD)] was seen in *n* = 11 (34%) cases, whereas *n* = 21 (66%) patients showed progressive disease (PD). In *n* = 9 patients surgery was performed subsequently in an individual therapeutic approach. Out of *n* = 5 patients receiving additional regional hyperthermia, *n* = 3 achieved PR or SD.

**Conclusions:**

This single-centre experience does also not support the promising phase Ib/II results for olaratumab/doxorubicin in STS. However, our findings do not preclude that olaratumab combination therapy could be valuable in a neoadjuvant setting. This warrants further exploration also taking into account the heterogeneous nature of STS.

## Background

Soft-tissue sarcomas (STS) are a rare and heterogeneous group of neoplasms of mesenchymal origin, which represent about 1% of malignancies in adulthood with an annual incidence rate in Germany of about 6 per 100,000 [1]. With over 50 different histologic subtypes, it remains difficult to establish a therapeutic standard. While many STS can be cured by surgery alone at an early stage of the disease, locally relapsing and metastatic disease continues to be a challenge and often requires multi-modal therapeutic approaches, especially in high-grade STS. In a palliative setting, single agent doxorubicin or doxorubicin combinations remain the standard of care for the majority of histologic STS subtypes [2]. Nevertheless, there is a high-unmet medical demand for improved STS treatment and innovative effective chemotherapeutic agents are needed.

Olaratumab is a human recombinant monoclonal immunoglobulin G subclass (IgG1) antibody, which binds specifically to platelet-derived growth factor receptor alpha (PDGFRA) and consecutively blocks ligand binding. As PDGFR signalling is known to be relevant in mesenchymal biology [3], a lot of hope was inspired within the STS community based on the promising results of a phase Ib/II trial showing a median progression-free survival (PFS) of 6.6 months in the olaratumab/doxorubicin arm compared to 4.1 months in the doxorubicin monotherapy cohort [4]. In accordance, olaratumab was approved in combination with doxorubicin by the FDA in 2016, especially as there was also an improved overall survival (OS) and overall response rate (ORR), which so far had not been documented for any other novel STS treatment. Hoping that this new approach would be paradigm changing, it seems that many patients have benefitted from the combination treatment since the approval of the drug. Surprisingly, the additional value of olaratumab in combination with doxorubicin treatment could recently not be confirmed in the large randomized, double-blind phase III ANNOUNCE trial [5].

While we were awaiting the detailed results of the ANNOUNCE trial, we concluded that our experience at the Charité–Universitätsmedizin Berlin, a large sarcoma centre, could further contribute to the understanding of these unanticipated efficacy results of olaratumab in STS.

## Methods

The aim of this retrospective analysis is to understand the real-world effectivity of the combination regimen of olaratumab/doxorubicin as measured by OS, PFS, and ORR (defined as the rate of patient achieving a CR, PR, or SD), and to evaluate the toxicity of the combination therapy.

**Fig. 1:**
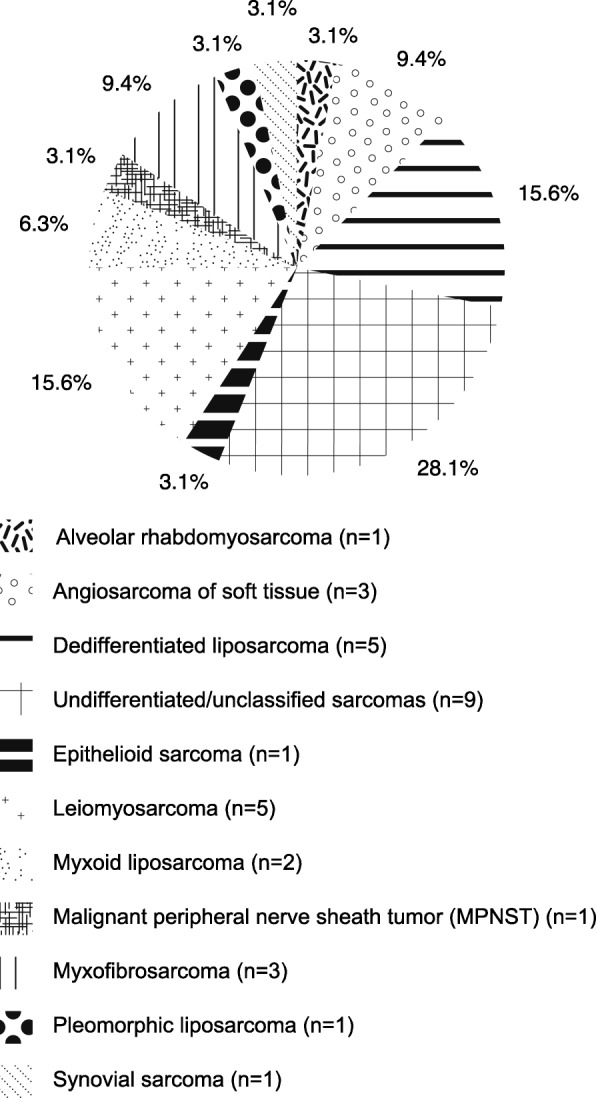
Histologic subtypes

We included a total of *n* = 32 STS patients who were all treated with olaratumab/doxorubicin at our institution between 2016 and 2019. Patients were included with institutional review board approval and patient informed consent in accordance with the local ethical guidelines. The majority of patients had either adipocytic sarcomas (*n* = 8), undifferentiated/unclassified sarcomas (*n* = 9), or smooth muscle tumours (*n* = 5). For detailed information about the histologic subtypes, see Fig. 1. The median patient age was 63 years (range 44–81) with *n* = 21 males and *n* = 11 females included. For detailed patients’ characteristics, see Table 1.

**Table 1:** Patients´ characteristics

Characteristic	n = 32
Sex n (%)	
male	21 (66)
female	11 (34)
Age (years)	
median	63
range	44–81
Stadium n (%)	
localized	10 (31)
metastasized	22 (69)
Grading n (%)	
G2, G3	22 (69)
G1	7 (22)
Gx^a^	3 (9)
Cycles of doxorubicin/olaratumab administered n (%)	
1 to 5	23 (72)
6 to 8	9 (28)
median no. of cycles	4
Exposure to doxorubicin	
median cumulative dose (mg/m^2^)	300
range (mg/m^2^)	75–600
Proportion of patients with delay of therapy due to toxicity/infection n (%)	4 (12,5)
Patients with previous treatment lines n (%)	
0	25 (78)
≥ 1	7 (32)
Response n (%)	
PR	4 (13)
SD	7 (22)
PD	21 (66)
Performance status (ECOG) n (%)	
0	14 (44)
1	16 (50)
2	2 (6)
Pattern of metastases n (%)	
lung only	3 (9)
multiple	6 (19)
Site of primary tumor n (%)	
extremity	8 (25)
retroperitoneum	8 (25)
trunc	14 (4)
head	1 (3)
uterus	1 (3)
Site of metastasis n (%)	
lung	6 (19)
liver	4 (13)
bone	2 (6)
other	7 (22)

*PR* partial remission; *SD* stable disease; *PR* progressive disease

^a^no formal grading available, but with clear histologic and radiologic features of high grade sarcoma

**Fig. 2:**
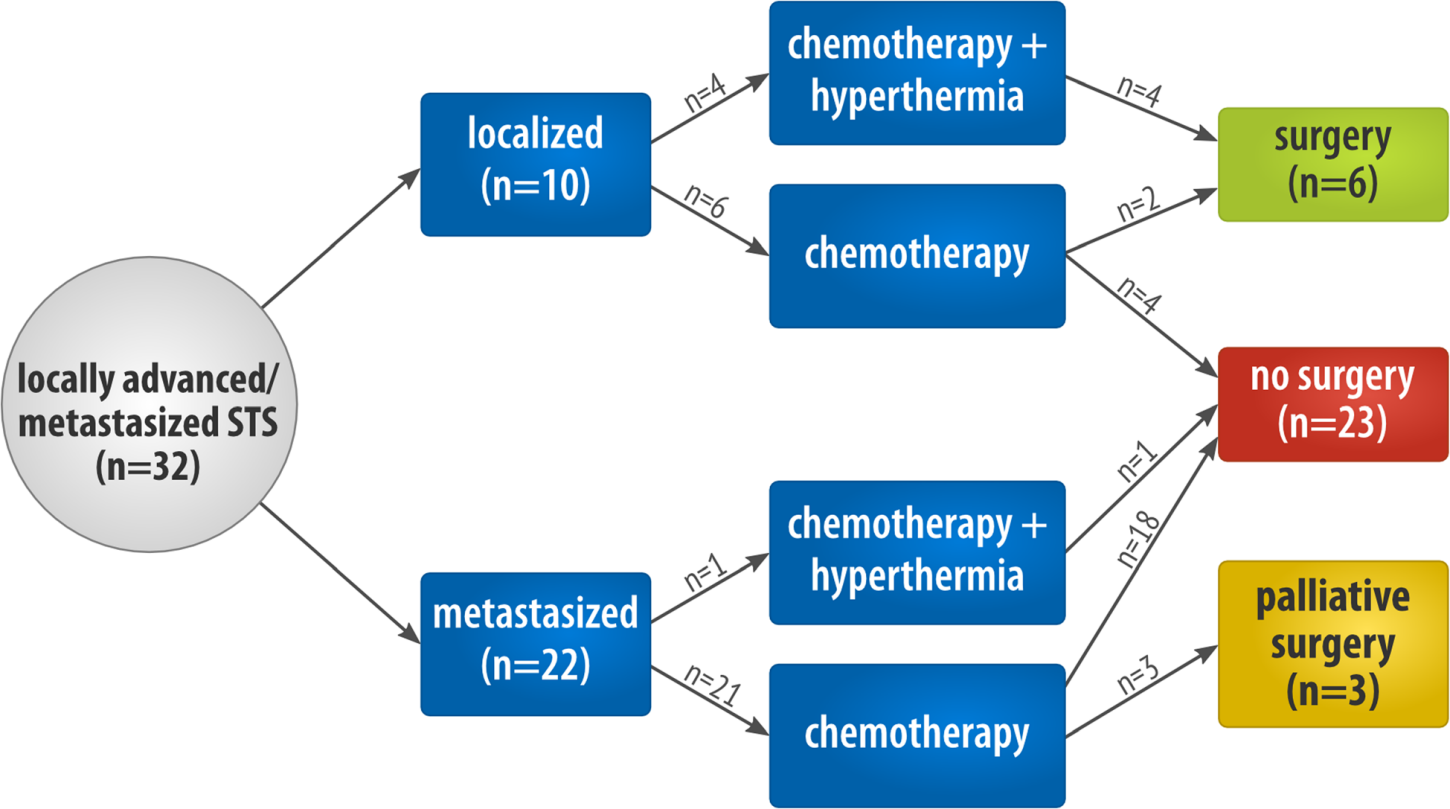
Detailed information on respective therapeutic sequence

Patients received olaratumab (15 mg/kg) intravenously on day 1 and day 8 plus doxorubicin (75 mg/m^2^) on day 1 of each 21-day cycle. All patients received olaratumab/doxorubicin in a palliative setting. In *n* = 25 of the patients it was given as first line therapy, and in *n* = 7 in more advanced treatment lines following trabectedin, pazopanib, paclitaxel, or other combination regimens. In *n* = 9 patients the systemic therapy was followed by surgery as a patient-adapted individual therapeutic approach. For detailed information on the respective therapeutic sequences, see Fig. 2.

In patients without clinical evidence for progressive disease, tumour response assessment was performed after 4 treatment cycles based on imaging with computed tomography (CT) or magnetic resonance imaging (MRI) scans. Response was evaluated in analogy to the RECIST v1.1 criteria. Follow-up analyses were performed every 3–4 months at physicians´ discretion. The median follow-up time in this analysis was 140 days (range 48–533). Toxicity was assessed according to the National Cancer Institute (NCI) criteria v5.0 at each visit.

## Results

### Response to therapy

Of the *n* = 32 STS patients treated with olaratumab/doxorubicin at our institution, patients completed on average 4 cycles (range 1–8), while *n* = 9 (28%) patients completed 6–8 cycles (see Table 1). In *n* = 5 of the patients with localized primarily inoperable disease, as assessed by the interdisciplinary tumour board, additional regional hyperthermia was given. This decision was made with the goal to possibly enable curative surgery in case of tumour response.

In our STS cohort, we observed a response in *n* = 11 (35%) cases (partial remission [PR] or stable disease [SD]). PR was seen in *n* = 4 patients (13%), *n* = 7 had SD (22%), whereas *n* = 21 had PD (66%) (see Table 2). The median progression-free survival (PFS) in the overall cohort was 3.1 months (range 0.6–16.2) and the median overall survival (OS) 4.6 months (range 1.6–17.5) (see Table 3).

**Table 2:** Overall response

	all patients	patients receiving surgery
outcome	n = 32	n = 9
PD n (%)	21 (65.6)	3 (33.3)
PR n (%)	4 (12.5)	4 (44.4)
SD n (%)	7 (21.9)	2 (22.2)

*PR* partial response; *SD* stable disease; *PR* progressive disease

Response was assessed based on imaging (CT or MRI) scans in analogy to the RECIST v1.1. criteria

**Table 3:** Survival rates

	all patients	patients receiving systemic therapy only	patients receiving surgical intervention
	*n* = 32	*n* = 23	*n* = 9
PFS median (range)	3.1 (0.6–16.2)	2.8 (0.6–16.2)	4.7 (2.1–11.9)
OS median (range)	4.6 (1.6–17.5)	3.9 (1.6–17.5)	7.4 (3.3–16.6)

*PFS* progression free survival; *OS* overall survival

A small number of n = 4 patients showed disease stabilization beyond 8 cycles of the combination regimen and received olaratumab maintenance therapy. In 3 out of 5 patients receiving additional regional hyperthermia a PR (*n* = 2), or SD (*n* = 1) was achieved.

### Surgical intervention post olaratumab/doxorubicin treatment

A small subset of our cohort (*n* = 9) underwent surgery following olaratumab/doxorubicin treatment (see Table 2). The majority of these patients demonstrated PR or SD (*n* = 6) while the remaining patients (*n* = 3) underwent a palliative resection despite PD due to the absence of other reasonable therapeutic options.

In the cohort of patients undergoing surgery (n = 9) we could observe the following differences compared to the cohort without subsequent surgery (*n* = 23): these patients did slightly less frequently show high grade G3 sarcoma (44% vs. 48%) and were less often treated in a metastatic setting (33% vs. 83%). The median PFS of this cohort was 4.7 months (range 2.1–11.9) compared to 2.8 months (range 0.6–16.2) in the cases without surgery. Similarly, the median OS was 7.4 months (range 3.3–16.6) for operated STS vs. 3.9 months (range 1.6–17.5), respectively (see Table 3). The number of patients who completed 6 to 8 cycles of doxorubicin/olaratumab was also higher in the cohort with surgery (44% vs. 26%, respectively).

### Treatment related toxicity

In our patient cohort, the combination treatment with olaratumab/doxorubicin was well tolerated and we did only observe few high-grade toxicities (grade 3 or higher). These consisted predominantly of hematologic toxicity (e.g. anaemia in *n* = 7 [22%] cases and leukopenia in *n* = 12 [38%] cases). Grade 3 or 4 febrile neutropenia or other infections occurred in 9% or 13% of the patients, respectively. Treatment discontinuation due to toxicity was only necessary in *n* = 1 (3%) of the patients.

We observed the following grade 1/2 toxicities: anaemia (*n* = 14 [69%]), leukopenia (*n* = 9 [28%]), decreased appetite (*n* = 10 [31%]), constipation (n = 7 [22%]), diarrhea (*n* = 2 [6%]), infections/infestations (*n* = 3 [9%]), musculoskeletal pain (n = 2 [6%]), abdominal pain (n = 1 [3%]), febrile neutropenia (n = 1 [3%]), infusion-related reaction (n = 1 [3%]), and pyrexia (n = 1 [3%]).

No toxicity was documented that clearly related to olaratumab treatment. Apart from infusion-related reactions (IRR), no distinct olaratumab-associated adverse events are known. We did not observe any higher grade IRR in our cohort. For the detailed overview on the toxicities during olaratumab/doxorubicin combination treatment see Table 4.

**Table 4:** Therapy-associated toxicity by grade per patient

Event	Any grade	Grade 3	Grade ≥ 4
Any toxicity n (%)			
Nausea Nausea	11 (34.4)	0 (0)	0 (0)
Fatigue	16 (50)	1 (3.1)	0 (0)
Neutropenia	15 (47)	2 (6.3)	10 (31.3)
Mucositis	6 (18.7)	1 (3.1)	0 (0)
Alopecia Alopecia	32 (100)	0 (0)	0 (0)
Vomiting	5 (15.6)	0 (0)	0 (0)
Anaemia Anaemia	29 (90.6)	7 (21.9)	0 (0)
Leukopenia Leukopenia	21 (65.6)	10 (31.3)	2 (6.3)
Constipation	7 (21.9)	0 (0)	0 (0)
Diarrhea Diarrhea	2 (6.3)	0 (0)	0 (0)
Decreased appetite Decreased appetite	10 (31.3)	0 (0)	0 (0)
Abdominal pain	3 (3.4)	1 (3.1)	1 (3.1)
Pyrexia	3 (9.4)	0 (0)	2 (6.3)
Musculoskeletal pain	2 (6.3)	0 (0)	0 (0)
Febrile neutropenia	4 (12.5)	1 (3.1)	2 (6.3)
Infections and infestations	7 (21.9)	1 (3.1)	3 (9.4)
Infusion-related reaction	1 (3.1)	0 (0)	0 (0)
Olaratumab-related toxicitiy	0 (0)	0 (0)	0 (0)
Toxicity leading to discontinuation	1 (3.1)	0 (0)	1 (3.1)
Cardiac dysfunction	0 (0)	0 (0)	0 (0)

Toxicity was assessed according to the National Cancer Institute (NCI) criteria v5.0

## Discussion

While the early results of the phase Ib/II trial with olaratumab/doxorubicin showed very promising clinical activity including a relevant improvement in OS [4], this could surprisingly not be confirmed in the large phase III ANNOUNCE trial [5].

In the phase Ib/II trial by Tap et al. the PFS and OS were considerably longer in the olaratumab/doxorubicin treated cohort compared to the monotherapy group (6.6 vs. 4.4 months and 26.5 vs. 14.7 months, respectively). Furthermore, the phase Ib/II study showed a disease control rate (CR, PR or SD) of 77.3% in the combination arm vs. 62.7% in the patients treated with single agent doxorubicin.

Astonishingly, the phase III ANNOUNCE trial presented at the ASCO annual meeting 2019 was not able to reproduce the additional therapeutic benefit of olaratumab in combination with doxorubicin. PFS in the doxorubicin plus placebo cohort was improved compared to the results of the combination arm, whereas concerning OS there was no difference shown between the two groups: 5.4 vs. 6.8 months and 19.7 vs. 20.4 months, respectively [5]. Disease control rate was also higher in patients receiving the monotherapy: 67.4% vs. 75.7%.

Similarly, our real-world data as well as a recently published Austrian analysis [6] did also not demonstrate the high disease stabilization rates seen in the phase Ib/II study, as SD was seen in only 22% of our cases compared to 59% of the phase Ib/II cohort and 54% in the ANNOUNCE trial. However, the rate of patients achieving a PR was similar with 13% compared to 15% in the phase Ib/II cohort and 13% in the ANNOUNCE trial, respectively.

The poorer disease stabilization and the shorter survival of our cohort may be caused by selection-bias. In our study, we mostly opted for olaratumab/doxorubicin if the patient did not appear to have an adequate performance status for the combination of doxorubicin and ifosfamide.

Concerning the patient characteristics “sex”, “age”, and “number of previous therapies” our patient population was in some respect more alike to the one of the phase III ANNOUNCE trial than to the phase Ib/II cohort. Doxorubicin and olaratumab was the first systemic therapy for the majority of patients treated in our centre (78%) and also in the ANNOUNCE trial (74%), in the phase Ib/II trial there were only 41% patients without previous therapy included.

In our study, there was a predominance of male patients (66%) whereas there were less male patients included in the phase Ib/II (44%) and in the ANNOUNCE trial (42%), respectively. Male sex has been shown to be an adverse prognostic factor in different oncologic diseases concerning outcome and response to chemotherapy [7–10]. There also were more male patients in the doxorubicin mono group of the phase Ib/II trial included, but not in the ANNOUNCE trial as there were 39% vs. 44% male patients receiving single agent and combination therapy, respectively. In summary, gender does not really help to distinguish the differences between the different treatment groups in the previously published data, but could in part explain the dismal results in our limited patient population.

In line with the interpretation of the results of the ANNOUNCE trial, possibly the most important factor influencing the efficacy of STS treatment in general, is the exposure to a certain dosage of anthracyclines. Our patients received a median number of 4 cycles doxorubicin compared to 6 cycles in ANNOUNCE trial and 7 cycles in the phase Ib/II trial. In our study, the combination regimen was well tolerated. We observed less toxicities than in the published data of the ANNOUNCE trial with only one exception: Hematologic toxicity (all grades) was more frequent in our cohort (anaemia 91% vs. 43% and leukopenia 66% vs. 32%, respectively) which could be due to the slightly older age of the patients included in our study (median 63 years vs. 57 years). In contrast to previously published results [4, 5], we did not notice any higher grade IRR, which is the most commonly described adverse treatment-related event of olaratumab. Additionally, there was no occurrence of therapy-limiting cardiac dysfunction, even though more than 25% of our patients completed 6 to 8 cycles of olaratumab/doxorubicin.

While our data do not support the initial enthusiasm on PDGFRA targeting, PDGFR signalling nevertheless plays a crucial role in oncogenesis as well as in angiogenesis and fibrogenesis [11–13]. As a result, this pathway has an impact on the tumour microenvironment, e.g. diffusion, and the growth of cancer cells [14]. Therefore, detailed efforts are undertaken to further understand the anti-tumour activity of PDGFR antibodies such as olaratumab. In preclinical xenograft studies, olaratumab was effective to inhibit cancer cell growth [15]. Olaratumab showed an inhibition of interstitial pressure followed by a better delivery of cytotoxic substances. Most likely, PDGFRΑ inhibition is not the only mode of action of olaratumab. Additionally, there seems to be a pre-sensitizing effect on the tumour stroma, which allows an increased tumour cell permeability [16].

Except for one patient with leiomyosarcoma treated with olaratumab monotherapy [17], data derived from other advanced malignancies show that there is nearly no therapeutic efficacy by the antibody alone [15, 17]. Altogether, olaratumab only seems to be effective in combination with cytostatic chemotherapy. Luckily, the addition of olaratumab to chemotherapy does usually not increase side effects as also demonstrated in advanced ovarian cancer and metastatic prostate cancer [18, 19].

As there is also some preclinical data showing a synergistic effect of olaratumab combined with doxorubicin in xenograft models of human rhabdomyosarcoma, it seemed reasonable to combine the monoclonal antibody with this established effective substance in the context of STS [2, 20, 21].

As for other combinatorial treatment strategies, one might speculate that combination with radiotherapy and/or hyperthermia could be beneficial. Combining a PDGFRA antibody with radiotherapy in a murine model was not proven successful, as Song et al. could not show a significant effect of olaratumab as a radiosensitizer. However, they did find a decrease of pulmonary micro metastases in mice treated additionally with the monoclonal antibody, which however was not significant [21]. Because of the frequent use of radiation therapy in an adjuvant setting, this question could have been addressed further in case of continued olaratumab availability.

So far, there is also no published data for the combination of olaratumab/doxorubicin with hyperthermia. The use of hyperthermia in addition to radiation therapy or chemotherapy is well established for STS. In 2018, Issels et al. published the final results of a multinational phase III trial exploring in STS the use of hyperthermia in combination with chemotherapy in the neoadjuvant setting. They could show a significant effect of additional hyperthermia on local PFS, DFS, and OS with an improvement of overall survival and local progression-free survival [22]. A retrospective analysis of the radio-oncologic department at the Charité–Universitätsmedizin Berlin also demonstrated a comparable therapeutic response of hyperthermia and radiation therapy in STS in a neoadjuvant setting with a reduced rate of surgical complications in the former group [23]. For that reason, in selected patients we combined doxorubicin/olaratumab with regional hyperthermia in the cohort of patients with borderline resectable localized tumours. While our results are preliminary, the data from our limited single-centre cohort demonstrates feasibility of a combination of anthracycline/olaratumab-based combination chemotherapy with hyperthermia in a neo-adjuvant setting. As a clinical benefit was seen in 3 out of 5 patients receiving this multimodal combination treatment, the potential application of a respective strategy clearly warrants further investigation in larger patient cohorts and might be especially beneficial in a neoadjuvant setting.

Thus, we urgently have to develop more effective therapies for advanced and metastatic STS, in addition to more reliable biomarkers that are needed to better predict tumour response. While the combination of olaratumab and doxorubicin might not be beneficial in all STS cases, certain subgroups might well benefit from the treatment. Unfortunately, different efforts could not establish PDGFRA as a reliable marker, as it is heterogeneously expressed in the stromal component of the tumour microenvironment and the tumour itself [24]. For instance, the ANNOUNCE trial showed an improved OS in patients negative for PDGFRA compared to those with relevant PDGFRA expression [5]. For the definition of subgroups possibly benefiting from olaratumab, it is vital to further explore the role of predictive biomarkers besides PDGFRA expression, which so far has shown no robust predictive value.

## Conclusions

The publication of the full results of the ANNOUNCE trial clearly demonstrate that we still do not fully understand the biology of STS and there remain many open questions. Our results from a single-centre cohort do also not support the high hopes that were put into an olaratumab/doxorubicin combination therapy based on the initial phase Ib/II trial data. While there may be an additional therapeutic effect of olaratumab for certain subgroups of patients with STS, e.g. in cases with less aggressive disease, we are convinced that one should also evaluate olaratumab/doxorubicin in the neoadjuvant setting in combination with hyperthermia. Additional real-world data would help to better understand the efficacy potential of olaratumab in different therapeutic settings in STS, and this could form the basis for additional studies even though the initial efforts have not been successful so far.

## Data Availability

Data supporting the results reported in the article are available from the authors upon reasonable request and with permission of Charité–Universitätsmedizin Berlin, corporate member of Freie Universität Berlin, Humboldt-Universität zu Berlin, and Berlin Institute of Health, Department of Hematology, Oncology, and Tumor Immunology, Campus Virchow-Klinikum, Berlin, Germany.

## Abbreviations

ASCO: American Society of Clinical Oncology
CR: Complete remission
CT: Computed tomography
DFS: Disease free survival
FDA: Food and Drug Administration
IgG: Immunoglobulin G
IRR: Infusion-related reactions
MRI: Magnetic resonance imaging
NCI: National Cancer Institute
ORR: Overall response rate
OS: Overall survival
PD: Progressive disease
PDGFRA: Platelet-derived growth factor receptor alpha
PFS: Progression-free survival
PR: Partial remission
SD: Stable disease
STS: Soft tissue sarcoma
